# A dataset on bathymetry and hydrology of an emerging periglacial lagoon in Svalbard, Arctic

**DOI:** 10.1016/j.dib.2025.111304

**Published:** 2025-01-14

**Authors:** Andrius Šiaulys, Aleksej Šaškov, Greta Kilmonaitė, Dzmitry Lukashanets, Tobia Politi, Aurelija Samuilovienė, Anastasija Zaiko, Sergej Olenin

**Affiliations:** a Marine Research Institute, Klaipėda University, H. Manto 84, 92294, Klaipėda, Lithuania; b Sequench Ltd., 1/131 Hardy Street, Nelson 7010, New Zealand

**Keywords:** Glacier retreat, Water mixing, Bottom relief, Halocline, Thermocline

## Abstract

In recent decades, the melting of glaciers has led to a consistent increase in the number of periglacial coastal lagoons that form in the place of receding glaciers in Svalbard, European Arctic. There is limited data on the geomorphology and hydrology of these novel formations, primarily because conducting research in remote polar regions is logistically challenging and expensive.

We present hydrological and bathymetric data collected in 2022-2024 in a newly formed lagoon located in the western part of Spitsbergen (Svalbard), between Eidembreen glacier and Eidembukta bay. The lagoon consists of several semi-isolated water bodies characterized by distinct bottom geomorphology and complex hydrological structure. The deepest part (42.8 m) was observed near the glacier front, while extensive shallow areas have depths of less than 1 m. Various hydrological parameters were measured during surveys (temperature, salinity, oxygen saturation, dissolved oxygen concentration, pH, photosynthetically active radiation, turbidity, conductivity, density, pressure and sound velocity), which indicate strong vertical stratification with pronounced halocline and thermocline in some parts of the lagoon.

The data collected will be utilized to analyze the physical and geographical conditions of the lagoon, as well as the distinguishing characteristics of its various sections, which vary in age by several decades. The data is essential for understanding the distribution patterns of biodiversity and the functioning of the lagoon ecosystem, which is the subject of a separate study. Furthermore, this data can be utilized to monitor and conduct comparative analysis of periglacial lagoons that are forming in various regions of Svalbard due to the climate change. This dataset can also be used to develop models of hydrological processes in periglacial water bodies of this nature, which arise from the interaction between a melting glacier and the influence of the sea.

Specifications Table

**Table:** 

Subject	Oceanography, Earth-Surface Processes, Global and Planetary Change, Hydrology and Water quality
Specific subject area	Hydrology of water bodies, Coastal geomorphology, Arctic environmental studies
Type of data	Processed data in excel and csv tables
Data collection	An inflatable boat Commando C5 with 5 hp engine was used to conduct bathymetric and hydrological studies in the lagoon. Bathymetry. Data on bathymetric profiles were collected using the Garmin Echomap UHD 92SV echosounder with GT56UHD-TM transducer over the course of three seasons: 2022, 2023, and 2024. This is an entry-level echosounder that does not have a motion sensor, so no motion compensation was applied to the collected data. The QPS Qimera software was utilized for the filtering of raw data. Additionally, in 2022 shallow depths were measured with a sonar Deeper chirp+2. Hydrology. The Valeport SWiFT SVP probe was used during the 2022 and 2024 surveys for measurements of pressure, sound velocity, temperature, salinity, density and conductivity at different depths. In 2023, a multiparameter probe CTD115M was used for measurements of pressure, temperature, conductivity, oxygen saturation, dissolved oxygen concentration, pH, photosynthetically active radiation, turbidity, salinity, density and sound velocity.
Data source location	Data were collected in a lagoon located in western part of Spitsbergen, Svalbard, European Arctic within these coordinates: • Northernmost point: 78°24’19,1’’N / 12°50’33,7’’E • Easternmost point: 78°22’10,4’’N / 12°55’44,8’’E • Southernmost point: 78°21’13,1’’N / 12°52’34,4’’E • Westernmost point: 78°22’30,8’’N / 12°45’26,8’’E Data are stored at the Marine Research Institute, Klaipeda University (Klaipeda, Lithuania)
Data accessibility	Repository name: Mendeley Data Data identification number: doi: 10.17632/zb9ngwk5vs.2 Direct URL to data: https://data.mendeley.com/datasets/zb9ngwk5vs/2
Related research article	-

## 1 Value of the Data

• This dataset provides information on bathymetry and hydrology of the novel periglacial lagoon that is forming as the glacier recedes in Svalbard, Arctic.
• The physical geography data are essential for biological/ecological studies on this lagoon that are conducted simultaneously.
• The data presented here can be utilized to monitoring and comparative analysis of periglacial lagoons which are becoming more prevalent in various regions of the Svalbard archipelago due to climate change and the subsequent interaction between melting glaciers and the adjacent sea.
• Using our data, it is possible to develop and calibrate a hydrological model of the lagoon. This could be a first step towards understanding the complex dynamics of this system and predicting its future trajectories in the context of intensifying global climate change.

## 2 Background

The retreat of glaciers and ice sheets in the Northern Hemisphere is a significant indicator of climate change [1]. The melting of glaciers leads to the formation of new Arctic coastal ecosystems, including periglacial lagoons [2]. Based on aerophotography, the Norwegian Polar Institute has discovered over 100 such recently emerging lagoons in Svalbard, urging for better understanding of the development and functioning of these coastal features [3]. The study area for this project is the lagoon next to Eidembukta Bay in West Spitsbergen. This location was selected due to the noticeable impacts of accelerated glacier retreat. As a result, a complex coastal water body has formed, connected to the retreating glacier on one side and separated from the sea by a narrow gravel spit on the other side. The data collected will enhance our comprehension of the hydrological processes in the lagoon, in relation to its geomorphological evolution. This data will serve as a foundation for future comprehensive investigations into the structure and functioning of this developing coastal ecosystem.

## 3 Data Description

The dataset consists of two directories: 1) “Bathymetry”, which includes files “depths_Deeper2022”, “depths_Garmin2022”, “depths_Garmin2023”, “depths_Garmin2024”, with indication to sonar used and the year of survey; 2) “Hydrology”, which contains three subfolders “2022”, “2023” and “2024” indicating the year of survey. Each subfolder consists of two files with coordinates (contains site names, longitude and latitude in WGS 1984 coordinate system) and hydrological data (contains hydrological data for each site in separate sheet). Data is provided both in .xlsx and .csv formats.

**Fig. 1:**
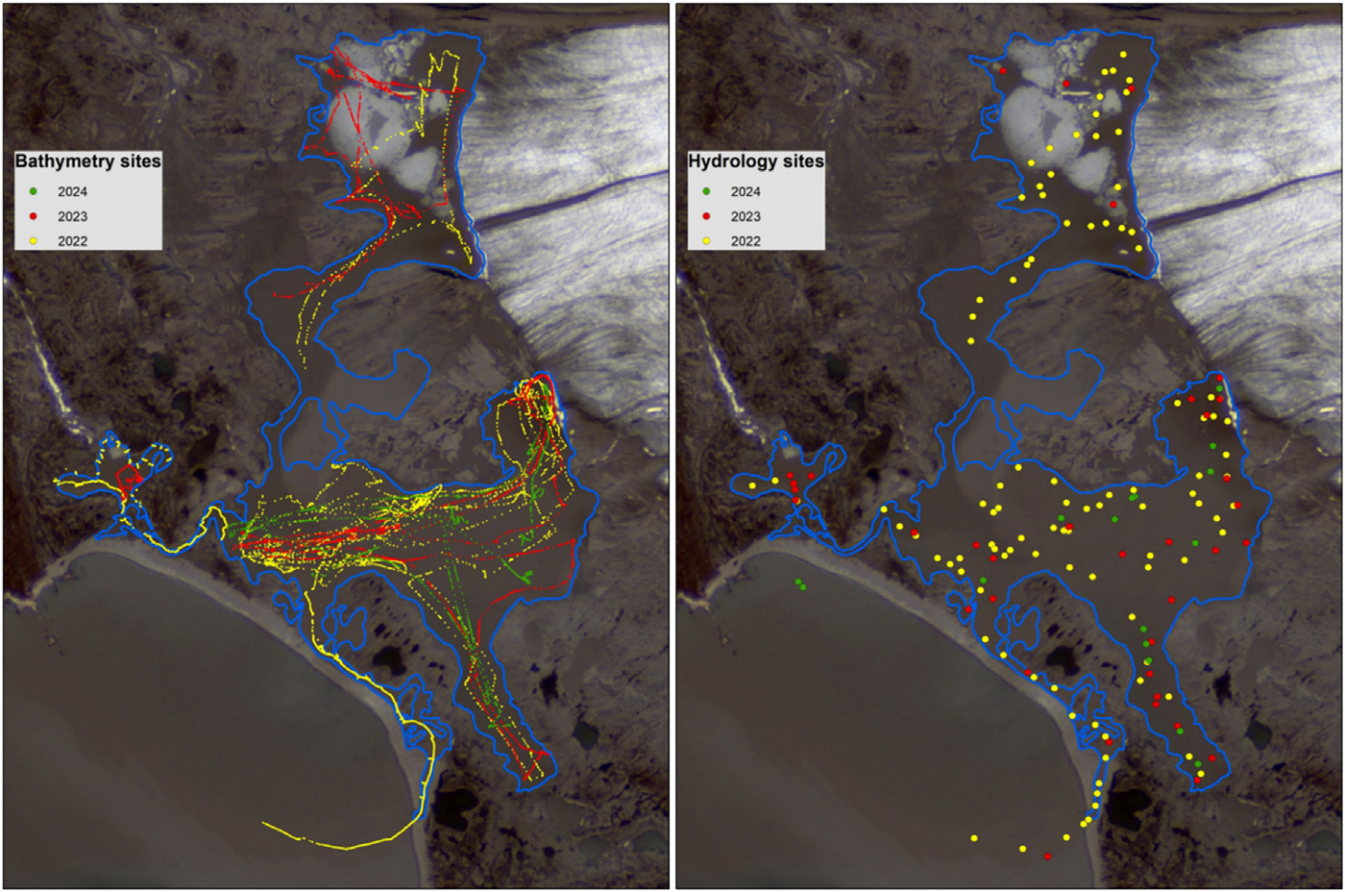
The location of bathymetry and hydrology sites during surveys in 2022, 2023 and 2024.

Bathymetrical data files consist of coordinates (longitude, latitude in WGS 1984 coordinate system) and depth measurements (in meters). Hydrological data from 2022 and 2024 consists of several parameters: depth profile (m), pressure (decibars), sound velocity (m s^-1^), temperature (degree Celsius), salinity (PSU), density (kg m^-3^), conductivity (milliSiemens per centimeter), date and time. Hydrological data from 2023 consists of following parameters: pressure (decibars), temperature (degree Celsius), conductivity (milliSiemens per centimeter), oxygen saturation (%), dissolved oxygen concentration (mg l^-1^), pH, photosynthetically active radiation (microeinsteins), turbidity (FTU), salinity (PSU), density (kg m^-3^), sound velocity (m s^-1^). The location of survey sites is presented in Fig. 1.

## 4 Experimental Design, Materials and Methods

Surveys were conducted in 2022 August 21-26th, 2023 August 14-19th and 2024 July 22-24th. An inflatable boat Commando C5 with 5 hp engine was used to conduct bathymetric and hydrological studies in the lagoon.

Bathymetry. The main instrument utilized for gathering bathymetry data throughout the 2022, 2023, and 2024 seasons was the Garmin Echomap UHD 92SV echosounder with GT56UHD-TM transducer. Each time the boat was deployed, the transducer was mounted on a pole below the keel. The raw Garmin data from each season of measurements was extracted and processed using QPS Qimera software. This software was used to remove false soundings and make overall improvements to the bathymetry grid. The Garmin data is collected using the WGS84 datum. Additionally, in 2022, for extremely shallow depths or areas inaccessible by boat, bathymetric data was collected using sonar Deeper chirp+2 mounted on a fishing rod or towed on the water surface close to the boat. Raw .csv data was extracted from “Fish Deeper” application, filtered and converted to excel and csv tables.

Hydrology. The Valeport SWiFT SVP probe was used during the 2022 and 2024 surveys to gather hydrological data. The Profiler comes with a range of integrated sensors, including GPS, pressure, temperature, and sound velocity. The calculation of density, salinity, and conductivity is derived from sound velocity and temperature through Valeport's proprietary DASH formula [4]. Raw .vp2 files were downloaded using “Valeport Ocean” application and converted into excel tables. In 2023, a multiparameter probe CTD115M was used for measurements of pressure, temperature, conductivity, oxygen saturation, dissolved oxygen concentration, pH, photosynthetically active radiation, turbidity, salinity, density, sound velocity. SST-SDA software was used to record and extract raw data, which was cleaned and converted into excel and csv tables.

All data is provided in .xlsx and .csv formats in Mendeley Data repository [5].

## Limitations

• Due to various factors that need to be taken into consideration, the bathymetric data may contain potential deviations and should be regarded as preliminary.
• During each deployment of the boat, there was an inevitable inconsistency in the mounting angles of the sonar. Additionally, there was no motion sensor to compensate for the movements of the sonar head. The system also lacked sound velocity profiles, which are crucial in a water body with a strong halocline that has been observed to shift from year to year. Furthermore, due to time constraints, bathymetric profiles were conducted alongside other tasks, such as plankton sampling, resulting in a lack of strict systematic grid organization in their distribution across the water area.
• The bathymetric and hydrological surveys did not cover the shallow areas of less than 1 m in depth, which make up a significant portion of the lagoon.
• The measurements were conducted exclusively in the summer months of July and August.

## Ethics Statement

The authors have read and follow the ethical requirements for publication in Data in Brief and confirming that the current work does not involve human subjects, animal experiments, or any data collected from social media platforms.

## Credit Author Statement

**Andrius Šiaulys:** Data conceptualization, Methodology, Investigation, Data curation, Writing-original draft, Writing-review & editing. **Aleksej Šaškov:** Methodology, Investigation, Software, Data curation. **Greta Kilmonaitė:** Investigation, Visualization. **Dzmitry Lukashanets & Tobia Politi:** Investigation, Writing: Reviewing and Editing; **Aurelija Samuilovienė & Anastasija Zaiko:** Writing: Reviewing and Editing. **Sergej Olenin:** Supervision, Investigation, Writing: Reviewing and Editing.

## Data Availability

Mendeley DataBathymetrical and hydrological data of newly formed lagoon near retreating Eidembreen glacier in Svalbard, Arctic (Original data).
